# *In vivo* imaging system for explants analysis—A new approach for assessment of cell transplantation effects in large animal models

**DOI:** 10.1371/journal.pone.0184588

**Published:** 2017-09-20

**Authors:** Weronika Zarychta-Wiśniewska, Anna Burdzinska, Radosław Zagozdzon, Bartosz Dybowski, Marta Butrym, Zdzisław Gajewski, Leszek Paczek

**Affiliations:** 1 Department of Immunology, Transplant Medicine and Internal Diseases, Transplantation Institute, Medical University of Warsaw, Warsaw, Poland; 2 Department of Clinical Immunology, Transplantation Institute, Medical University of Warsaw, Warsaw, Poland; 3 Department of Bioinformatics, Institute of Biochemistry and Biophysics, Polish Academy of Sciences, Warsaw, Poland; 4 Department of Urology, Medical University of Warsaw, Warsaw, Poland; 5 Division of Dermatology and Venereology, Department of Clinical Sciences, Lund University, Lund, Sweden; 6 Department of Large Animal Diseases with Clinic, Veterinary Research Centre and Center for Biomedical Research, Faculty of Veterinary Medicine, Warsaw University of Life Sciences (WULS–SGGW), Warsaw, Poland; Faculty of Animal Sciences and Food Engineering, University of São Paulo, BRAZIL

## Abstract

**Introduction:**

Despite spectacular progress in cellular transplantology, there are still many concerns about the fate of transplanted cells. More preclinical studies are needed, especially on large animal models, to bridge the translational gap between basic research and the clinic. Herein, we propose a novel approach in analysis of cell transplantation effects in large animals explants using *in vivo* imaging system (IVIS®) or similar equipment.

**Material and methods:**

In the *in vitro* experiment cells labeled with fluorescent membrane dyes: DID (far red) or PKH26 (orange) were visualized with IVIS®. The correlation between the fluorescence signal and cell number with or without addition of minced muscle tissue was calculated. In the e*x vivo* study urethras obtained from goats after intraurethral cells (n = 9) or PBS (n = 4) injections were divided into 0.5 cm cross-slices and analyzed by using IVIS®. Automatic algorithm followed or not by manual setup was used to separate specific dye signal from tissue autofluorescence. The results were verified by systematic microscopic analysis of standard 10 μm specimens prepared from slices before and after immunohistochemical staining. Comparison of obtained data was performed using diagnostic test function.

**Results:**

Fluorescence signal strength in IVIS® was directly proportional to the number of cells regardless of the dye used and detectable for minimum 0.25x10^6^ of cells. DID-derived signal was much less affected by the background signal in comparison to PKH26 in *in vitro* test. Using the IVIS® to scan explants in defined arrangement resulted in precise localization of DID but not PKH26 positive spots. Microscopic analysis of histological specimens confirmed the specificity (89%) and sensitivity (80%) of IVIS® assessment relative to DID dye. The procedure enabled successful immunohistochemical staining of specimens derived from analyzed slices.

**Conclusions:**

The IVIS® system under appropriate conditions of visualization and analysis can be used as a method for *ex vivo* evaluation of cell transplantation effects. Presented protocol allows for evaluation of cell delivery precision rate, enables semi-quantitative assessment of signal, preselects material for further analysis without interfering with the tissue properties. Far red dyes are appropriate fluorophores to cell labeling for this application.

## Introduction

Cellular transplantology is one of the most dynamically developing fields in medicine and cell therapy procedures are becoming a clinical practice in increasing number of applications. However, there are still many concerns regarding the fate of grafted cells, the safety and efficacy of this kind of treatment. Therefore, there is a general agreement that more preclinical data are needed to rationally expand the scope of applications for cell therapy. Studies on large animals are especially desirable as they fill the gap between rodent models and humans allowing for more precise prediction if certain therapy can be effective after translation to the clinic [1]. Large mammalian species have been successfully used in testing cell transplantation effects in many different applications like cardiovascular diseases [2], osteochondral defects [3], neural disorders [4] or urinary incontinence [5]. The objectives of preclinical studies in the field of cell therapy are usually: i) the assessment of functional effect, and ii) describing the fate of grafted cells which encompasses parameters like cell survival, migration from delivery site, graft differentiation and integration with the host tissue. Evaluation of cell fate after transplantation in large mammalian species is a very demanding task. Currently, the most commonly methods used to assess the cellular graft survival are: i) quantitative or semi-quantitative analysis of graft amount in the homogenates of the whole target area [6], and ii) histological analysis of serial tissue sections [7]. The first method is achieved by an examination of graft specific RNA or protein expression, which allows for estimation of graft survival in the certain time point. However, this technique makes impossible the parallel assessment of structure and location of a graft and its integrity with the host tissue. On the other hand, the histological method of tissue analysis does not allow for quantitative assessment of transplanted cell survival. Moreover, the sectioning and analysis of the whole target area in large animals is very laborious, cost- and time-consuming. Those difficulties in verifying cell transfer effects constitute a significant restriction in large animal model studies in which the number of animals per group is usually small (determined by the high cost, logistical difficulties as well as ethical considerations). Moreover, none of described methods enables the evaluation of the injections precision rate, while the accuracy of cell delivery was recognized as one of crucial aspects conditioning the efficacy of this therapy [8, 9]. Therefore, the objective of this study was to develop a protocol which would improve and simplify current methods in assessment of cell transplantation effects in studies involving large animals.

We propose a novel approach in analysis on the basis of intraurethral cell transfer. This method assumes the two-step protocol. First step is to screen the whole area of interest with the *in vivo* imaging system (IVIS®) or equivalent equipment in order to detect the signal from labeled cells, and if possible, to quantify the signal. The second step is the typical histological analysis of tissue cross-sections, but performed only on preselected areas chosen during the first screening step.

The IVIS® or other similar systems, which read bioluminescence and fluorescence are dedicated to do research on small animals such as mice or rats. The use of spectra distribution algorithms allows high specificity imaging and minimizing the impact of autofluorescence. Those systems enable intravital assessment of different parameters, at various time points, following the nature of changes. We hypothesized that implementation of IVIS® for screening of large animals-derived explants would make the evaluation of grafted cell fate less time-consuming, labor-intensive, would allow gaining more data from one sample comparing to current approaches and enables the assessment of cell delivery precision. According to our knowledge, the available literature does not describe this kind of utilization of IVIS® or equivalent systems.

The aim of the study was to determine whether the IVIS® is suitable as a method for *ex vivo* imaging of isolated organs after cell transfer. The secondary aim was to compare the usefulness of membrane dyes in an orange and far red range of fluorescence for the use in IVIS® in large animal-derived explants. We tested this hypothesis by using caprine urethras isolated after autologous transplantation of cells labeled with two different fluorochromes.

## Materials and methods

To achieve the objectives, the study was divided into two stages: 1) *in vitro* part and 2) *ex vivo* part.

1. *In vitro* study was performed to determine: a) the correlation between the strength of fluorescence and the number of cells for the chosen dyes and b) the impact of tissue autofluorescence on the reading of specific signal derived from studied fluorochromes.

2. *Ex vivo* study on isolated urethras in order to determine: a) the presence of the specific, fluorophores-derived signal in the whole explanted urethras using IVIS®, b) the presence of transplanted cells by analyzing tissue sections using fluorescence microscopy, c) the compatibility of the results obtained by these two methods.

### *In vitro* study

The material for this part constituted primary bone-marrow mesenchymal stem cells (BM-MSC) and myoblasts isolated from adult goats. The use of merged population is linked to the hypothesis regarding the beneficial effect of these two cell types co-transplantation which was recently presented by our group [10]. The procedures of cell isolation and culture were previously described [11]. The cells originated from animals which were designated to control group in another experiment. This study was carried out in strict accordance with the recommendations in the Guide for the Care and Use of Laboratory Animals of the National Institutes of Health. The protocol was approved by the Local Ethics Animal Welfare Commission (Permit number: 39/2012). The animals were purchased from goat herd nr PL2000013. During the experiment, goats were maintained with constant access to food and water in groups of 4–6 animals. They could move freely in pens of appropriate dimensions. The biopsy procedures were performed in anesthetized goats. Animals were sedated with 0.4 mg/kg of midazolam intramuscularly (Midanium®, Polfa Warszawa S.A.), followed by intravenous administration of propofol in bolus (2–4 mg/kg b.w. depending on the reaction, Propofol, Scanofol®, ScanVet). The anesthesia was maintained with isoflurane (2%, Aerrane, Baxter Polska). Fentanyl was used as analgesic agent.

#### Cells staining and preparation of dilution series

Cells after 4^th^ passage were harvested, counted and stained with one of membrane fluorescent dyes: DID or PKH26. The dyes and staining details were as following:

- DiLC18(5)-DS (DID) [1,1'-Dioctadecyl-3,3,3,-tetrametlylindodicarbocyanine-5,5-disulfonic acid], Ex = 650 nm, Em = 670 nm (AAT Bioquest); the concentration of 10μM, in PBS, 15 min, at 37°C, with continuous stirring on roller mixer.
- PKH26, Ex = 551 nm, Em = 567 nm (Sigma-Aldrich); the concentration of 5μM, a solution in Diluent C provided by manufacturer, 4 min. at RT.

A total of 38 million cells were used (19 million per dye) for the procedure. The dyes concentrations were selected based on the initial optimization. After labeling procedure the cell number and mortality was evaluated (Trypan blue test).

Cells were suspended in PBS and a dilution series were prepared for each dye: 2x10^6^, 1x10^6^, 0.5x10^6^, 0.25x10^6^, 0.125x10^6^, 0.0625x10^6^, 0.03125x10^6^/100 μl of PBS. PBS solution was used as a blank test.

#### Cell analysis by using the IVIS®–*in vitro* study

A 96-well plate with black walls and bottom (BD Falcon Microtest Black 96 Well Plate) was used in order to inhibit influence of background fluorescence during analysis. In 48 wells an equal amount of minced poultry muscle tissue was placed. In the remaining wells cell suspensions were placed in decreasing dilution in triplicates by applying 100 μl/ well (plate layout is presented in S1 Fig). Measurements were made using the IVIS Spectrum system (Caliper Life Sciences). For the DID dye excitation wavelength was 640 nm and emission wavelength successively 680, 700, 720, 740, 760 and 780 nm. For PKH26 excitation wavelength was 535 nm and emission wavelength 580, 600, 620, 640, 660 nm. Cell suspensions were then transferred into corresponding wells with muscle tissue by pipetting them inside the minced tissue (S1 Fig). The aim of this procedure was to mimic the situation when cells stained with membrane fluorescent dyes are evaluated after transplantation into muscle tissue. This combination of muscle tissue and cell suspension was analyzed by using the IVIS® at the same wavelengths for each dye listed above. The analysis was performed by using Living Image 4.4. software (Caliper Life Sciences). An automated spectral distribution algorithm (called *Spectral unmixing*) was used. This kind of analysis should separate the specific signal originating from a particular fluorochrome and the background signal originating from the autofluorescence and food. The fluorescence specific signal was shown as the Radiant Efficiency (Emission light [photons/sec/cm^2^/str]/ Excitation light [μW/cm^2^]. Fluorescence signal for each individual well was counted by selecting region of interest (ROI) and quantifying as the Total Radiant Efficiency (TRE, [photons/sec]/[μW/cm^2^]). TRE represents the sums of fluorescent pixels within the ROI. For each number of cells the mean value was calculated. The correlation between the average TRE and number of cells was calculated using Microsoft Office Excel 2007. Signal-to-background ratio was calculated for both fluorochromes (ROI2—ROI1) / ROI1 where ROI1 is a measurement of the tissue fluorescence without the addition of cells, and ROI2 the reading for the cells in minced muscle tissue. Calculations were made for the same wells of the meat before and after the addition of the cell suspension.

### *Ex vivo* study

Material for this part of the study constituted urethras collected from 13 euthanized female goats. Animals were euthanized 28 days after either autologous intraurethral cell transplantation or intraurethral PBS injection. Transplanted cells were labeled, suspended in PBS (total volume 400 μl) and administered into the urethral wall with 2 million of cells/injection. Each injection volume was 50 μl. Some goats underwent transplantation with DID labeled cells only (n = 4), whereas the other group (n = 5) underwent transplantation with either DID stained cells or PKH26 stained cells (separated depots at the same time points). The grafted cells were either BM-MSC, myoblasts or combination of those two populations, however in this experiment only the type of staining and not the cell type was a differentiating factor. The remaining 4 goats were injected with PBS only.

The collected material was divided as following:

- material from experimental groups (injected with cells):

CELL_DID_ = 9 urethras injected with DID labeled cells (analysis with parameters for DID dye)

CELL_PKH26_ = 5 urethras injected with PK26 labeled cells (analysis with parameters for PKH26 dye)

- material from the control group (injected with PBS solution):

PBS_DID_ = 4 urethras (analysis with parameters for DID dye)

PBS_PKH26_ = 3 urethras (analysis with parameters for PKH26 dye)

#### Collection and preparation of tissue for analysis

**Fig 1:**
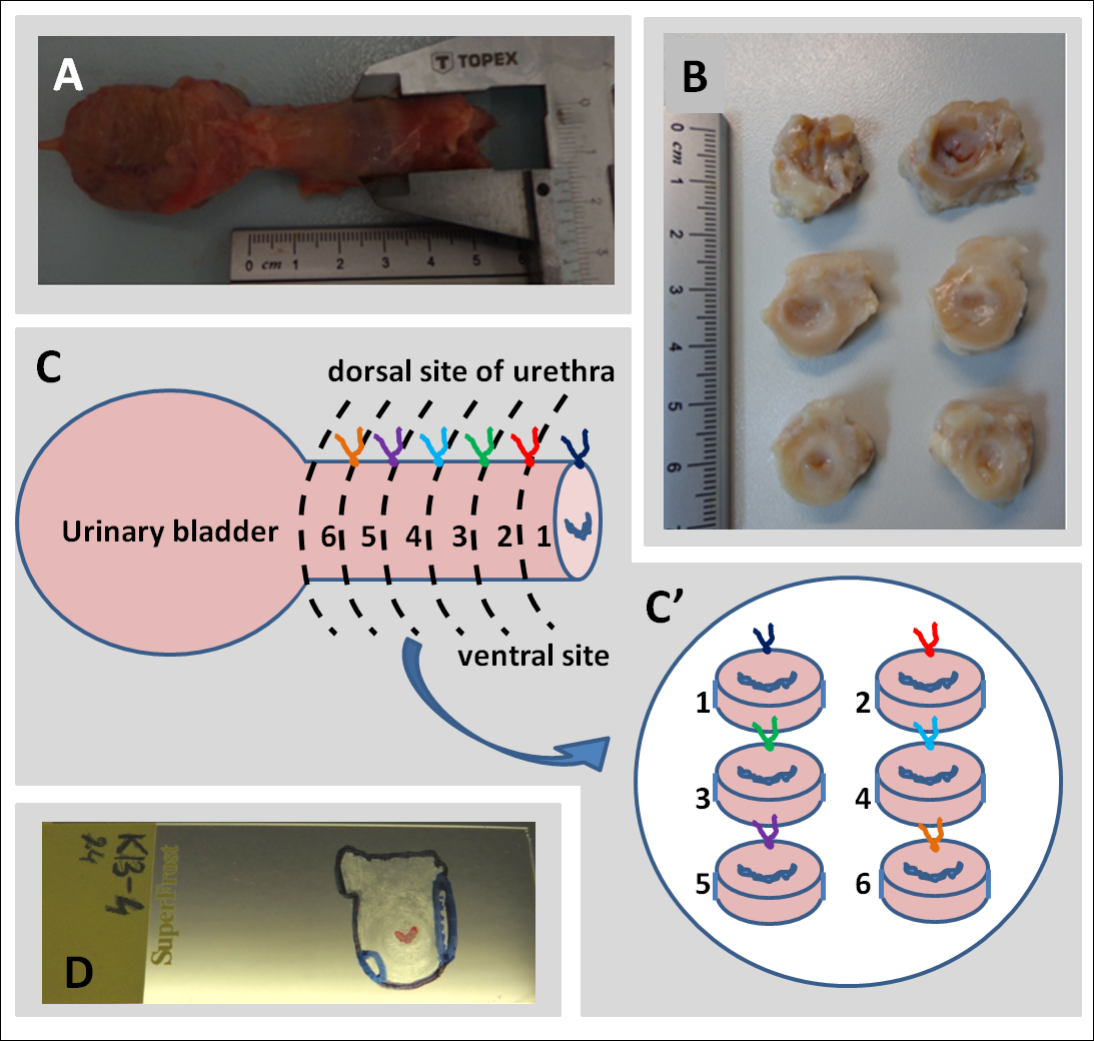
Preparation of posthumously collected urethra for analysis in *ex vivo* experiment. A) Photograph of freshly isolated urethra with bladder and a method of length and diameter measuring; B) Slices of whole urethra for analysis with IVIS®; C) Schematic arrangement of tissue slices for IVIS® analysis. caudal–top, dorsal–upwards; D) Photograph of the microscopic preparation with drawn model "map" after fluorescence microscopy analysis. Lines shows the outline of the preparation (black), urethra light (red), location of spots showing specific fluorescence (blue).

At the end of the experiment, whole urethras with bladders were posthumously collected from animals. The length and diameter of the urethras were evaluated and documented (Fig 1A). The tissues were placed in 4% (w/v) *p*-formaldehyde in PBS, pH = 7.25 (Sigma-Aldrich). The samples were rinsed for three consecutive days in PBS (at a frequency of once per day), and then placed in a solution of 18% (w/v) sucrose (Sigma-Aldrich), which acts as a cryoprotectant. The material was stored at 4°C until analyzed using the IVIS®, and then frozen at the temperature -80°C.

#### *Ex vivo* visualization of fluorescence by using IVIS®

The urethras were cut into cross-slices of about 0.5 cm thickness. As female caprine urethra is 3–4 cm long, such a cutting resulted in 5–7 fragments. (Fig 1B). Prepared slices were laid on a Petri dish always in the same arrangement: caudal side of the slice on the top and the dorsal side of the urethra upwards (Fig 1C and 1C'). This system allowed for subsequent identification of the right, left, dorsal and ventral sides of the slice according to the *in vivo* orientation. *Ex vivo* imaging was performed using IVIS® for respective fluorochromes (DID and/ or PKH26) at the same wavelengths as in *in vitro* experiment. The data from the readings were analyzed by using the Living Image 4.4 software. Two methods of visualization were used:

1. proposed by the manufacturer for specific fluorochromes resulting from the application of automatic algorithm division of spectra called spectral unmixing. The algorithm was used to determine the relative contribution from each fluorophore for every pixel of the image. In the assumption, this procedure allows to show a specific fluorescence signal derived from the transplanted cells and avoids the visualization of the background signal (originating from the food and tissue autofluorescence).
2. resulting from the manual setting ranges of minimum and maximum fluorescence strength. The ranges were selected on the basis of control (PBS injected) urethras images and *in vitro* experiments on cells, guided by the principle that in the control urethras fluorescence appropriate for the reading fluorophore is not visible (background fluorescence extinction, based on the initial optimization for this application).

#### Microscopic examination of collected urethras

Frozen slices of the urethras were cut into cross-sections of about 10μm thickness using cryotome (MICROM HM 525, Microm). From each fragment of the urethra, at least 10 sections on different depth of the slice were cut. This gave a 50–70 sections from each urethra which underwent microscopic evaluation. Any manipulation on the cross-sections, including the fixation, resulted in a decline of the fluorescence strength of both the PKH26 and the DID dyes, so the location of fluorescent points was identified on raw sections immediately after thawing. The entire sections were viewed by fluorescence microscopy using the appropriate filter (Olympus IX51). The analysis aimed to 1) detect spots with specific fluorescence and 2) determine the location of the fluorescent areas on the section "map" (Fig 1D). Fluorescent spots, defined as specific, had to be repeated on the several subsequent sections and showing no signal in other filters. This allowed for the elimination of artifacts caused e.g. by the folded tissue, which is seen as enhanced, but non-specific signal under a fluorescence microscope.

To verify if the visible fluorescence comes from the cells, randomly selected sections (¼ fragments were examined in this way) were stained with a DAPI solution at working concentration of 0.5 mg/ml, incubation time 4 min., RT. After washing, the preparations were coated with a mowiol solution (Sigma-Aldrich) and covered with the cover slip. Stained sections were analyzed using a fluorescent microscope Olympus IX51 and CellSens™ microscope imaging software or with slide scanner Axio Scan.Z1 (Zeiss) and Zen Blue second edition software (Zeiss).

Furthermore, some sections were designated for immunohistochemical staining. This analysis was performed to confirm the possibility of precise identification of both the location of the injected cells and their differentiation. Sections were fixed and permeabilized for 10 min in acetone, temp. -20°C. They were surrounded by a hydrophobic barrier and blocked with 1% (w/v) Bovine Serum Albumin (Sigma-Aldrich) + 5% (v/v) Normal Donkey Serum (Sigma-Aldrich) in PBS for 30 min, RT. They were incubated with the primary antibodies diluted in blocking solution for 90 min, RT. Anti-desmin antibody (Monoclonal Mouse Anti-Human Desmin, DakoCytomation) was applied in a concentration of 1:30. After washing, samples were probed with a secondary antibody conjugated with a green fluorochrome Alexa Fluor 488 (Ex = 493 nm, Em = 519 nm; Jackson ImmunoResearch) in concentration of 1:100, 60 min, RT. Specimens were washed again and nuclei were stained with DAPI as described above. Visualization was performed with a fluorescence microscope (Olympus IX51). Microscopic analysis of sections was made independently, without implying the images created with the IVIS®.

#### Verification of compatibility between results from *ex vivo* imaging using the IVIS® and microscopic examination

Data from IVIS® and microscopy were recorded in the 0/1 score (any specific fluorescence present = 1, absent = 0, for each of urethra slices). This analysis did not distinguished numbers of spots within a slice.

The comparison of data obtained from the IVIS® as a “diagnostic test” and microscopic evaluation as a “gold standard” was performed using diagnostic test function (PQStat software v.1.6.2), where a pair constituted the results from both types of analyzes in 0/1 score for the same tissue fragment. Sensitivity, specificity, accuracy of test, positive and negative predictive value as well as positive and negative likehood ratio were rated.

## Results

### *In vitro* part

#### Cell staining for *in vitro* assay

**Fig 2:**
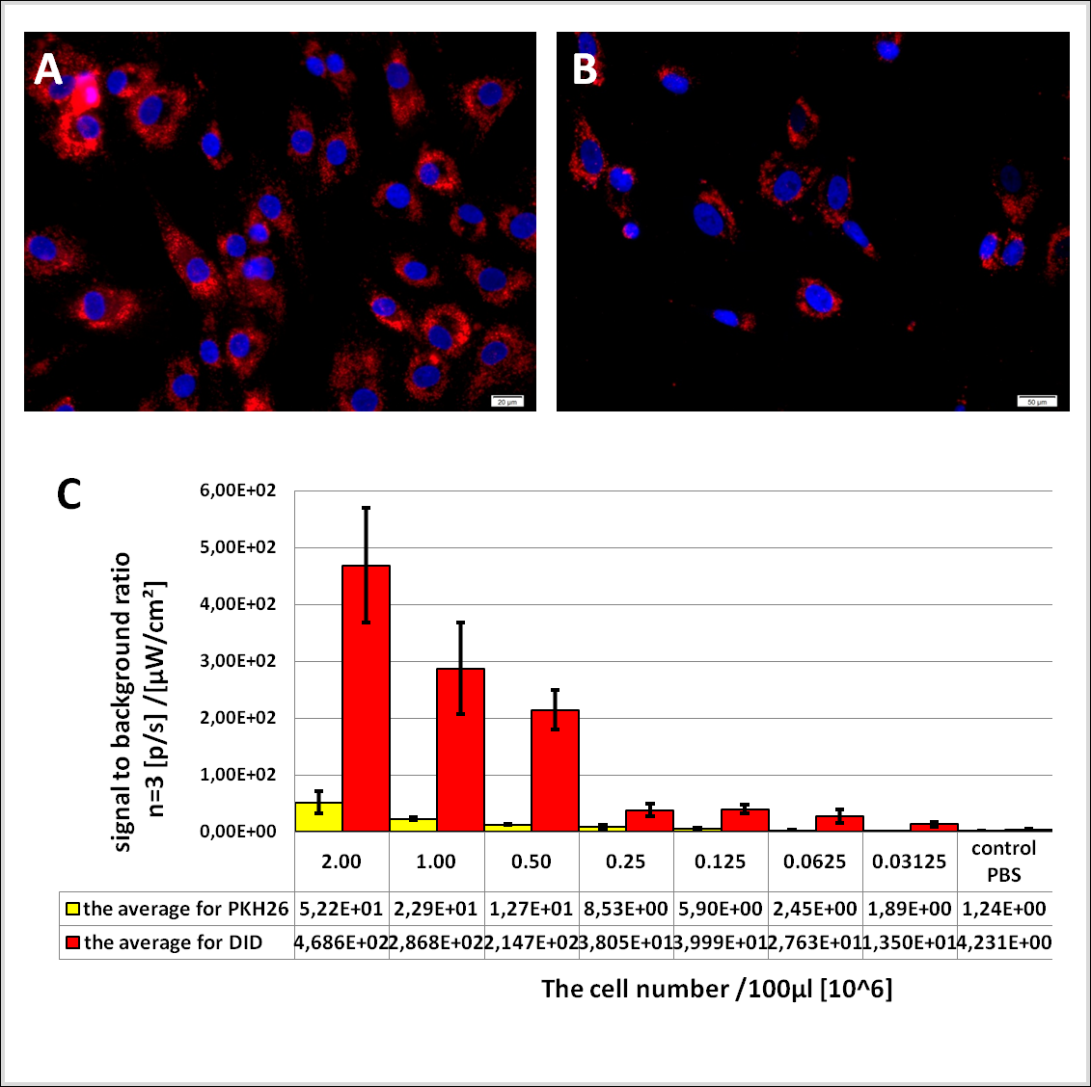
Evaluation of the cell staining effectiveness and strength of fluorescence signal for the transplantation procedure and *in vitro* experiment. Fluorescence microscopy pictures: A) cells labeled with PKH 26 (red fluorescence), B) cells labeled with DID (red fluorescence). Nuclei were labeled with DAPI (blue). C) Graph showing strength of fluorescence signal from PKH26 (yellow) and DID (red) labeled cells relative to background fluorescence. The measurements were performed for subsequent decreasing number of cells in triplicates. Data presented as averages +/- standard deviations.

Short-term effectiveness of staining was close to 100% (Fig 2A and 2B). The mortality of cells stained with PKH26 was 10%, and the mortality of cells labeled with DID amounted 12%. For the experiment 13 million stained cells for each of the fluorochromes were used.

#### Analysis of cell fluorescence in *in vitro* experiment using the IVIS®

For each of tested fluorochrome the spectral efficiency of labeled cells (in a range of tested cells numbers) per surface unit was determined. The signal strength for both fluorochromes was decreasing proportionally to the decreasing number of cells per well. For cells stained with DID image provided by the IVIS® was visible in wells containing more than 0.25x10^6^ cells. Less than 0.125x10^6^ of cells per well did not provide a visual effect after spectral unmixing analysis. The visible fluorescence faded away with the number of 0.25–0.125x10^6^ cells per well. Similarly, PKH26-derived signal was disappearing in wells with 0.25–0.125x10^6^ of cells. However, the radiant efficiency and the min-max pixel intensities represented by pseudo-color bars were different for both tested fluorochromes (S1A and S1B Fig). Next, readings for the cells transferred to the wells with minced muscle tissue were performed and fluorescence strength was evaluated for each fluorochrome (S1A' and S1B' Fig). Similarly to the previous experiment, for DID the visual effect was no longer visible in wells with less than 0.125x10^6^ cells while for PKH26 signal disappeared already for a number of cells less than 0.25x10^6^. Signal to background ratio for both dyes is directly proportional to the number of cells, however, for DID the values are much higher than for PKH26 what indicate that DID-derived signal is distinctly less affected by the background in comparison to PKH26 (Fig 2C).

### *Ex vivo* part

#### Imaging the urethras with the IVIS® to detect fluorescence from the DID dye

**Fig 3:**
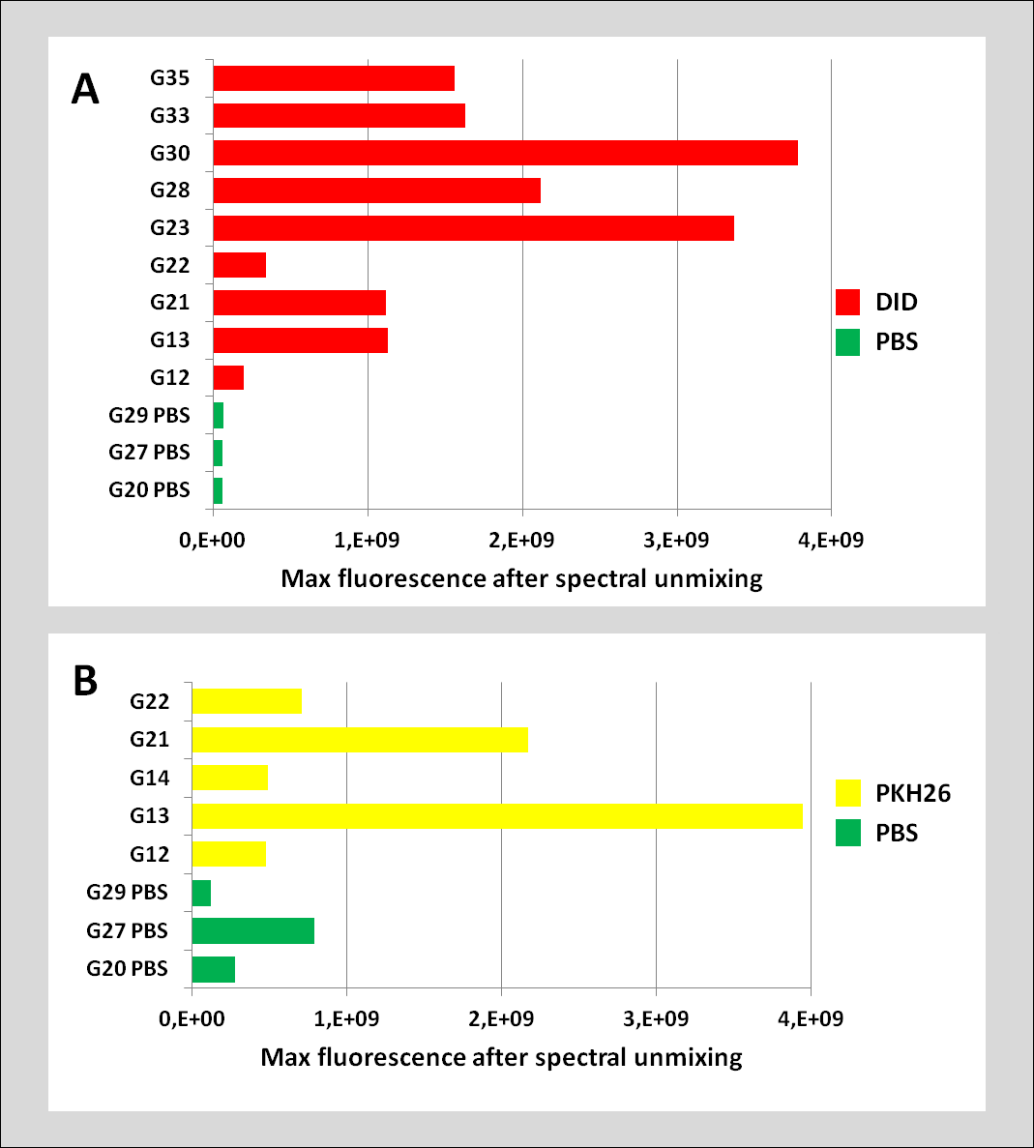
The comparison of fluorescence signal originating from transplanted and control urethras read by IVIS®. The bar graphs indicating maximal strength of fluorescence signal from DID (red, A) and PKH26 (yellow, B) transplanted urethras compared to the control group (green). Data obtained after spectral unmixing algorithm application. The ranges of the min-max fluorescence intensity, were selected automatically by the program.

**Fig 4:**
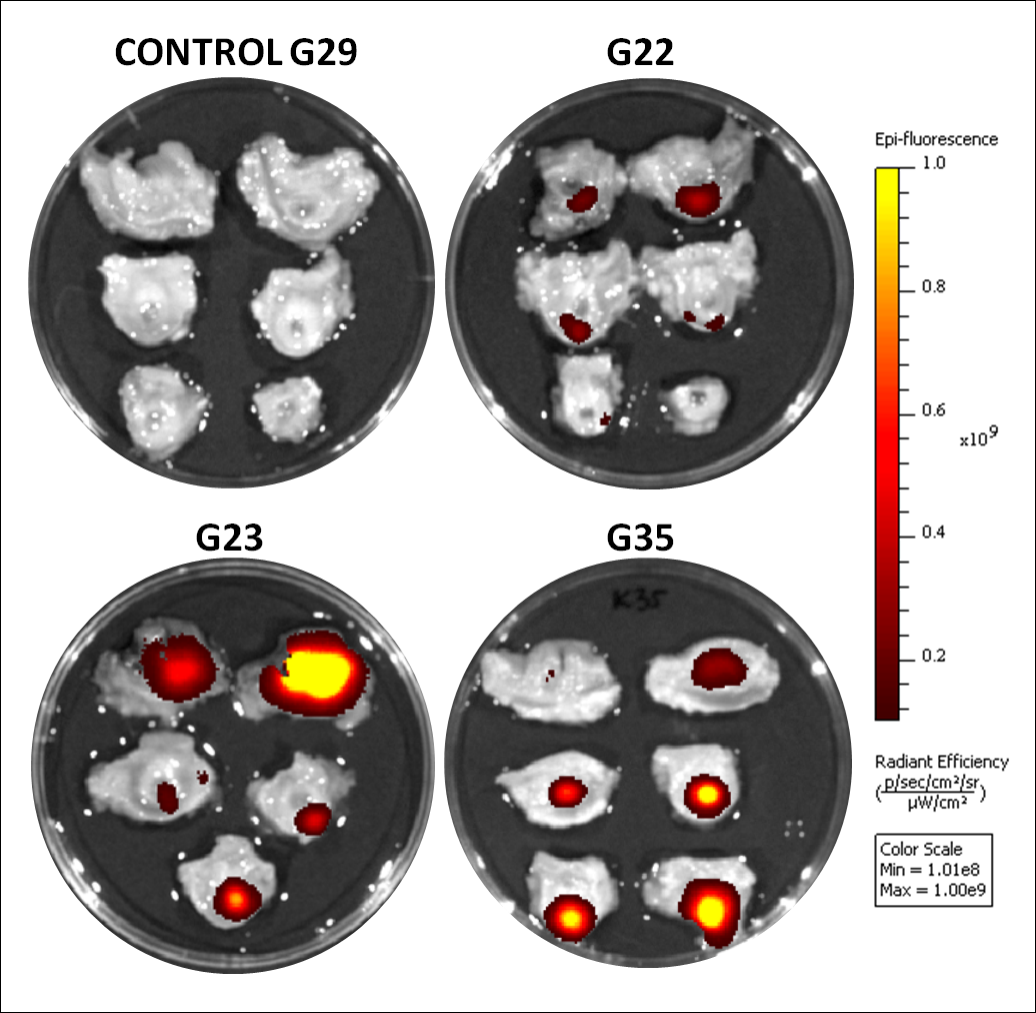
Images of representative urethras from control and experimental groups analyzed with IVIS® for the presence of DID labeled cells obtained by manual alignment of the spectrum. The color scale bar shows the range of strongest to weakest signal (1.01x10^8^- 1.00x10^9^). The intensity is strongest for the yellow colored points. The darker the spot, the weaker the signal; Ex. G22- experimental goat number.

First, the spectral unmixing algorithm was used. The ranges of the min-max fluorescence intensity, which were selected automatically by the program, differed for respective samples. The maximum tissue fluorescence in control samples was similar, in the range of 5x10^7^- 6.3x10^7^ and the maximum fluorescence of the tissues from the CELL_DID_ group was at least 2x10^8^, and in 7 out of 9 of the urethras in the test group exceeds 1x10^9^ (Fig 3A). Therefore it was clear that the signal from the transplanted urethras is higher than controls. However, the visible colored spots in PBS_DID_ samples suggested the presence of cells labeled with DID, while it was known that this tissue came from animals that were injected only with the PBS solution. This result indicated that this type of analysis is not satisfactory in the case of unknown samples testing. Moreover, in this type of analysis the color scale scope, illustrating the intensity of radiation, was different for respective samples, preventing the quick, indicative semi-quantitative comparison of the analyzed samples. Therefore, it became necessary to perform an additional manual selection of the visible fluorescence range. Such a unified scale for all goats analyzed for the presence of DID was chosen based on observations from the *in vitro* experiment. The image lower threshold (Min.) was increased in order to eliminate visible signal from all negative control samples (PBS from *in vitro* experiment and tissues from PBS_DID_ group) and not to lose the signal from DID positive control (cells from *in vitro* experiment). This kind of manual setting for the DID allowed to achieve a picture in which the fluorescence from all negative controls was invisible (pixels intensity is below minimum threshold on the color scale bar) while in the examined tissues from CELL_DID_ group the points indicating the specific fluorescence were visible (Fig 4). Moreover, the use of the same range (min-max scale) allowed the approximate comparative assessment of the signal (both in terms of the intensity and the area) in the CELL_DID_ samples, which should reflect the number of transplanted cells in these tissues. It was then determined on a 0/1 system for each urethral slice the presence of specific fluorescence for the two types of tested samples analysis: the automatic algorithm and manual settings (Tables 1 and 2). It was found that in 6 of 54 slices from experimental CELL_DID_ group score differed (11%) between the two types of reading. This result indicated that the use of manual reading range allows semi-quantitative analysis of the studied samples, but at the same time changing the reading in the 0/1 system. Microscopic examination had to decide which result is closer to the truth.

**Table 1:** The presence of specific DID fluorescence in urethras from study group after IVIS® spectral unmixing algorithm application.

Goat number	urethra slice number						
	I	II	III	IV	V	VI	VII
G20 PBS	1	1	1	1	1	1	NS
G27 PBS	1	1	1	1	1	1	NS
G29 PBS	1	1	1	1	1	1	NS
G12	1	1	1	1	0	1	NS
G13	0	0	1	1	0	0	NS
G21	0	1	1	1	1	1	NS
G22	1	1	1	1	1	0	NS
G23	1	1	1	1	1	NS	NS
G28	0	1	1	1	0	0	NS
G30	0	1	1	1	0	1	1
G33	0	1	1	1	1	0	NS
G35	0	1	1	1	1	1	NS

„1" indicates the presence and „0'' lack of registered fluorescence signal. Urethra slices in which fluorescence is reported are shaded orange. NS—no slice with this number.

**Table 2:** The presence of specific DID fluorescence in urethras from study group after manual alignment to the control in IVIS®.

Goat number	urethra slice number						
	I	II	III	IV	V	VI	VII
G20 PBS	0	0	0	0	0	0	NS
G27 PBS	0	0	0	0	0	0	NS
G29 PBS	0	0	0	0	0	0	NS
G12	0	0	1	1	0	0	NS
G13	0	0	1	1	0	0	NS
G21	0	1	1	1	1	1	NS
G22	1	1	1	1	1	0	NS
G23	1	1	1	1	1	NS	NS
G28	1	1	1	1	1	0	NS
G30	0	1	1	0	0	1	1
G33	0	1	1	1	1	0	NS
G35	0	1	1	1	1	1	NS

„1" indicates the presence and „0'' lack of registered fluorescence signal. Urethra slices in which fluorescence is reported are shaded orange. Heavy gridlines indicates differences between outcome from IVIS® automatic spectral unmixing algorithm application and manual ranges setting. NS—no slice with this number.

#### Microscopic examination of sections for the presence of fluorescence derived from DID

In order to verify the results obtained from IVIS® analysis, we performed systematic microscopic analysis of sections prepared from examined urethras' fragments. At least 10 raw cross-sections for each slice of all tested urethras were carefully examined. As a result, 45 of 54 slices contained the transplanted cells labeled with DID. The comparison of the microscopic analysis and data from IVIS® imaging with manual setting ranges were assessed with the 0/1 system (absent/ present). To assess the specificity and sensitivity of the IVIS® method the fluorescence microscopy was admitted as a reference technique. Based on this assumption, 1 false positive result and 9 false negative results was recorded in IVIS® analysis in comparison to the microscopic data (Tables 3 and 4). Therefore, the DID positive points indicated in tissue explants by IVIS® system will be in high probability the actual locations of the DID marked graft. At the same time, this analysis shows that the IVIS® may not detect part of the specific points (approximately 20%).

**Table 3:** Contingency table. Scheme.

frequencies observed		reality (gold standard)			
		transplanted cells present (+)	transplanted cells absent (-)	sum	
diagnostic test	positive result (+)	TP	FP	TP+FP	PPV
	negative result (-)	FN	TN	FN+TN	NPV
	sum	TP+FN	FP+TN	n = TP+FP+FN+TN	
		Sensitivity	Specificity		

TP- true positive; FP- false positive; FN- false negative; TN- true negative; PPV- positive predictive value; NPV- negative predictive value.

**Table 4:** Contingency table for DID dye.

frequencies observed		reality (microscopic analysis)		
		transplanted cells present (+)	transplanted cells absent (-)	sum
diagnostic test (IVIS analysis)	positive result (+)	36	1	37
	negative result (-)	9	8	17
	sum	45	9	54

Microscopic examination was assumed as a reference method. Method using IVIS® constituted a diagnostic test. The comparison of the microscopic analysis and data from IVIS® imaging with manual setting ranges. This analysis did not distinguished number of spots within an urethra slice and only the presence/absence of transplanted cells in the particular slice registered by microscopy or IVIS®.

Microscopic observations indicated that the points which were observed in the microscope, and were not present in the IVIS® image were relatively small fluorescent spots. Additionally, the assessment of DID positive points location within the fragment revealed that spots visualized with IVIS® and confirmed by microscopy were in each case coinciding.

#### Imaging the urethra using IVIS® to detect fluorescence from the PKH26 dye

As in the case of DID fluorochrome, first the spectral unmixing algorithm was applied. The result was the presence of visible color spots in all analyzed fragments, both experimental and control. The maximum tissue fluorescence in PBS_PKH26_ samples was distinctly higher than in PBS_DID_. Two of three control urethras (G20 and G29) were analyzed both for PKH26 and DID dye. The maximal fluorescence measured for PKH26 in G20 amounted 7.8x10^8^ and 1.1x10^8^ in G29 (Fig 3B), whereas for the same urethras the max fluorescence for DID amounted 5.2x10^7^ and 6.2x10^7^ respectively (Fig 3A). Trials to suppress the visible signal to the level adequate to control were undertaken as it was done for DID. The aim was to eliminate the visual spots in all control samples and, at the same time, to keep the signal coming from PKH26 positive control (cells from *in vitro* experiment). Such a correction was successfully performed for DID dye, however it turned out to be impossible for PKH26 fluorochrome. The reason for this failure was too strong and diverse signal in control samples. Therefore, a range which eliminated visible signal from all control samples resulted in lack of signal in any of studied samples. Trails to determine the scope, which could be used as a reference point for all samples failed. The results of analyzes in different variants of an example urethra are presented in Table 5. Finally, the level of fluorescence was selected for each of the goats from the study group separately, based on an assessment of the initial reading (the image obtained before the separation of spectra) and the effects of the application of the spectral unmixing algorithm.

**Table 5:** Settings difficulties for PKH26.

goat number	G35	unmix	different manual settings			alignment to control G29	alignment to control G20	alignment to control G11
**urethra slice number**	**I**	1	1	0	0	1	0	1
	**II**	1	0	0	0	1	0	1
	**III**	1	1	0	0	1	0	1
	**IV**	1	1	1	0	1	0	1
	**V**	1	1	0	0	1	0	1
	**VI**	1	1	0	0	1	0	1

Comparison of different IVIS® settings application influence on the fluorescence visible to the recipients eye on the example of the G35 urethra. „1" indicates visible signal, „0” the lack of a visible signal. Urethra slices in which fluorescence is reported are shaded orange.

#### Microscopic examination of sections for the presence of PKH26 fluorescence

Similarly, as in the case of DID fluorochrome, IVIS® analysis was verified using a fluorescent microscope. For each urethra slice at least 10 raw sections were accessed. Classification of the point as positive was the same as in the DID case. The analysis revealed that 11 of 31 slices (35.48%) contained the transplanted cells labeled with PKH26. Due to the problems presented above in establishing a satisfactory method of reading the PKH26 dye, independent analyzes for three different types of reading presentation were performed: 1) immediately after automatic spectral unmixing, 2) after an additional manual adjusting ranges individually for each urethra and 3) after an additional alignment to control G29 ranges for all samples equally. The results obtained after spectral unmixing and alignment to G29 control were the same, therefore they are presented as one version in the Table 6. The results obtained after applying manual settings are presented in the Table 7. Twenty false positive results were recorded in IVIS® analysis using spectral unmixing algorithm and alignment to G29 in comparison to the microscopic data. This constitutes 65% of analyzed slices. Analysis of manual min-max ranges resulted in obtaining false positive results in 12 slices of the 31 analyzed. Therefore, the detection of true positives points using such assumptions is highly unlikely. It was also very difficult to confirm the true negative points using different settings. The number of slices without PKH26 points in microscopic analysis amounted 20 while for various IVIS® settings was 0, 0 or 7.

**Table 6:** Contingency table for PKH26 dye obtained after applying spectral unmixing and alignment to G29 control in IVIS®.

frequencies observed		reality (microscopic analysis)		
		transplanted cells present (+)	transplanted cells absent (-)	sum
diagnostic test (IVIS analysis)	positive result (+)	11	20	31
	negative result (-)	0	0	0
	sum	11	20	31

Microscopic examination was assumed as a reference method. Method using IVIS® constituted a diagnostic test. The set of microscopic analysis of raw sections for the presence of the transplanted cells labeled with PKH26. This analysis did not distinguished number of spots within an urethra slice and only the presence/absence of transplanted cells in the particular slice registered by microscopy or IVIS®.

**Table 7:** Contingency table for PKH26 dye obtained after applying manual settings in IVIS®.

frequencies observed		reality (microscopic analysis)		
		transplanted cells present (+)	transplanted cells absent (-)	sum
diagnostic test (IVIS analysis)	positive result (+)	9	13	22
	negative result (-)	2	7	9
	sum	11	20	31

Microscopic examination was assumed as a reference method. Method using IVIS® constituted a diagnostic test. The set of microscopic analysis of raw sections for the presence of the transplanted cells labeled with PKH26. This analysis did not distinguished number of spots within an urethra slice and only the presence/absence of transplanted cells in the particular slice registered by microscopy or IVIS®.

#### Statistical analysis

**Fig 5:**
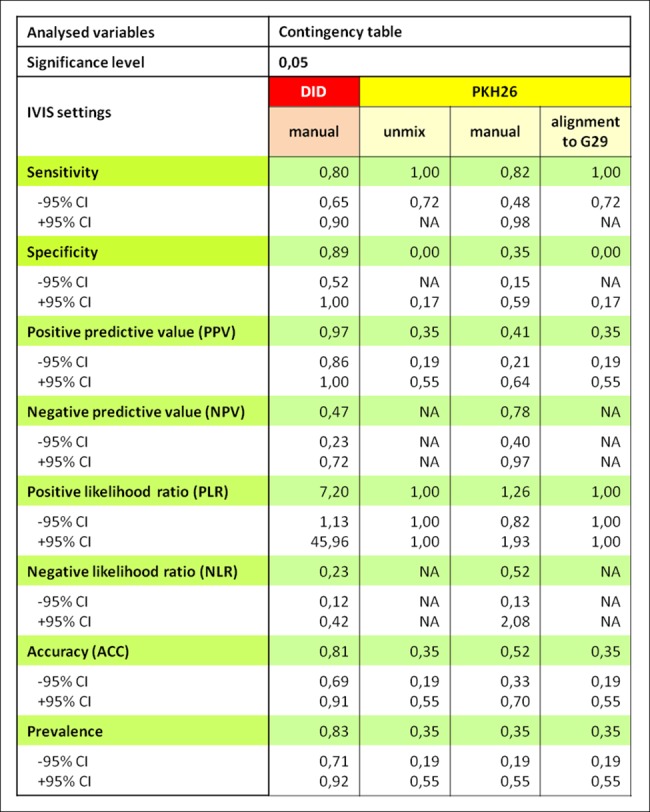
Evaluation of the diagnostic test reliability based on the contingency tables. Microscopic examination was assumed as a reference method. Method using IVIS® constituted a diagnostic test. The comparison of data obtained from the IVIS® and microscopy was performed using diagnostic test function (PQStat software v.1.6.2).

For DID 80% of the slices containing the transplanted cells has been properly qualified (were positive in IVIS®) while for PKH26, depending on the analysis method, it was 82% for manual settings or 100% for spectral unmixing algorithm or alignment to G29. In the DID case together with a high sensitivity goes high specificity as 89% of the slices containing no grafted cells labeled with DID has been properly qualified (were negative in IVIS®). IVIS® method cannot be considered as specific for PKH26 with the adopted in presented herein assumptions (0 or 35% respectively). A slice that received a positive IVIS® result at 97% contained the transplanted cells labeled with DID and for PKH26 it was true only for 35% or up to 41% of cases. A slice that received a negative IVIS® result at 47% contained no transplanted cells labeled with DID. This value could be estimated only for manual IVIS® settings for PKH26 (78%); Fragment analyzed in IVIS® (regardless of the result) will be properly classified in 81% of cases for DID. For PKH26 the best method was the manual settings, but even then we could only expect the correct classification of 52% of analyzed samples. Results presented in this paragraph are summarized in Fig 5.

#### Microscopic evaluation of sections from tissue after cells transplantation, stained for the presence of nuclei

**Fig 6:**
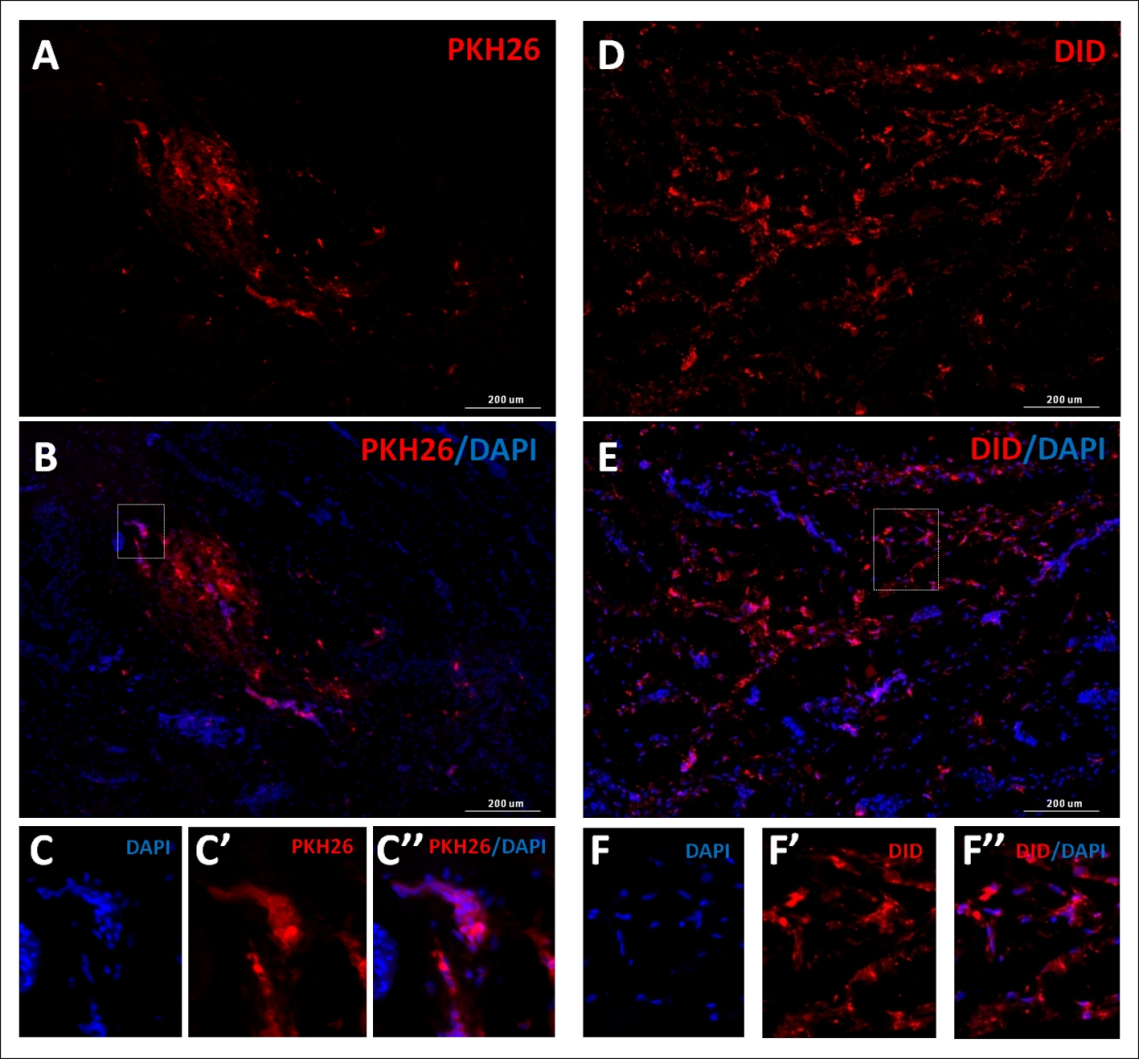
Scans of the urethra cross-sections. Urethras after transplantation with PKH26 (A, B) or DID (C, D) labeled cells. Images in columns represent the same field of view: A, D) the fluorescence derived from the membrane dye (the red fluorescence in both cases), B, E) membrane dyes and cell nuclei stained with DAPI- blue fluorescence. Scale: 200 μm. The rectangular areas marked on images B and E are shown enlarged below- C and F respectively. C, C', C" and F, F', F" represent the same field of view.

In order to confirm that the microscopically localized color spots were cells, staining of nuclei with DAPI was performed. This experiment confirmed that previously located structures were cells stained with fluorochromes used for the experiments with healthy nuclei, showing no degradation characteristics. Representative staining effect is shown in Fig 6.

#### Immunohistochemical staining

**Fig 7:**
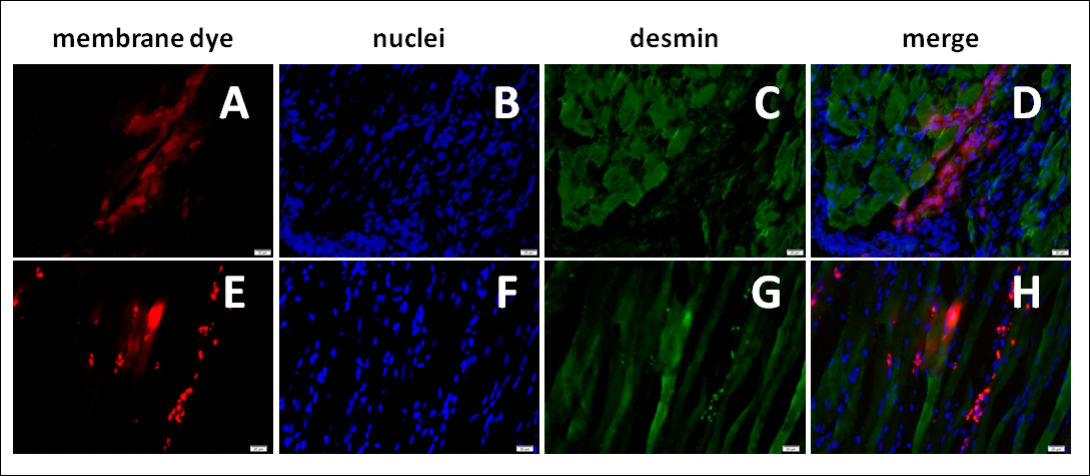
Effects of exemplary immunohistochemical staining for the presence of desmin. Micrographs of the urethra cross-sections after the transplantation of cells stained with DID (upper panel) and PKH26 (lower panel). Images in rows show the same field of view, wherein A, E illustrate the fluorescence derived from the membrane dye (the red fluorescence in both cases), B, F- DAPI stained cell nuclei, C, G- desmin labeled with antibody conjugated with a green fluorochrome AF488, D and H merged images. Scale: 20 μm.

Selected sections derived from fragments positive for the presence of labeled cells were subjected to immunohistochemical staining procedure. Desmin was visualized with IHC method. This procedure allowed for the precise localization of the transplanted cells in relation to the muscular layers of the urethra and to assess the differentiation of transplanted cells in the muscle structures (Fig 7). Obtained results demonstrate that a preliminary IVIS® analysis enables further research procedures on the same parts of the tissue.

## Discussion

The aim of this study was to define the novel protocol for assessment of cell transplantation effects in large experimental animals (including parameters like graft survival, integration and differentiation as well as injection precision). Our hypothesis was that IVIS® or similar equipment could be used for analysis of tissue explants in terms of cell presence and localization.

This study was performed on caprine primary cells and urethras collected after autologous cell transplantation. Urinary incontinence is a highly prevalent clinical condition in which cellular therapy has been proposed to correct urethral sphincters deficiency [8, 9, 12–14]. The problem of intraurethral cell delivery precision have been recognized by many groups of researchers [15–17]. The aim of intraurethral cell therapy is to administrate the agent precisely into urethral muscle layer with minimal invasiveness what is technically very demanding. Optimization of such a delivery method can be successfully carried out only on large experimental animals. The present study was performed on goats, because recently published description of caprine female urethra anatomy suggests that goat is a superior animal for testing intraurethral cell transfer efficacy than pigs or dogs [11].

At the stage of preclinical studies, each of the methods requires confirmation of the injection accuracy in the target tissue. In order to identify transplanted cells, they must be labeled in advance. Labeling must be relatively stable—at least during the observation period, non-toxic, easily visualized and specific. In the case of simultaneous use of several cells types, different markers to distinguish various populations in the analyzed material are required. The use of membrane dyes meets the above requirements and is now a popular method for tracking cells after transplantation [18–20], especially in autologous systems where the donor and recipient are genetically the same [21].

As two cell populations were used for transplantation in this study, it was necessary to use at least two markers to track the cells fate. The procedure of labeling with the membrane dyes is quick, labeling efficiency is close to 100%, toxicity is low and the effects of procedure are repeatable. Dyes of green fluorescence was not taken into account due to well known strong autofluorescence of tissue particularly pronounced within this range of wavelengths [22–24]. Therefore dyes of orange and far red fluorescence range were selected. Membrane dye PKH26 was previously used for cell transplantation therapies in the field of stress urinary incontinence by various research groups [17, 25–27]. It was shown that PKH26 does not adversely affect cell proliferation and cell morphology [28] and that cells labeled in this way were seen in the tissue 4 months after grafting [29]. Presented herein results indicate however that this marker has limited usability for visualization by IVIS®. Tissue autofluorescence makes it difficult to set the range that reflects the real fluorescence strength even after the spectra separation. Only for a very high concentration of the dye, the signal in a spot is so clear (bright yellow color in the IVIS® image) that can be distinguished from non-specific light expressed with dark red color. The inability to determine the scope of reference in turn does not allow to conclude with a high degree of certainty about the presence of transplanted cells in the tissue only based on IVIS® imaging. When trial is blinded, the distinction of tissue from study and control groups based on the IVIS® analysis is problematic or even impossible. Another limitation associated with PKH26 use regards its diffusion on neighboring structures. Li et al. [30] demonstrated that PKH26-labeled cell fragments added to the unstained cells *in vitro* or injected intravenously caused that unstained cells became PKH26-positive in both the *in vitro* and *in vivo* analysis.

Another tested fluorophore was DID. It belongs to the family of far red dyes. The reading of far red wavelengths is much less burdened with the background or the autofluorescence effect. DID is used in the experiments utilizing intravitally imaging to observe the transplanted cells fate [31, 32]. It was confirmed that this dye does not adversely affect the MSC properties *in vivo* and *in vitro* such as proliferation and differentiation capacity or ROS and cytokine production, which enhances to use it for preclinical testing of cell therapy effects [33]. Honig and Hume [34] demonstrated that DID, in contrast to PKH26, does not diffuse between adjacent cells. However, in more recent study Lassailly et al. [35] demonstrated that labeling with dyes Dil, DiD, DIR or PKH26 causes the appearance of micro environmental contamination, even if the used dye concentration is lower than recommended. It was concluded that microparticles spread both by the direct contact between cells and through by diffusing from cell surface to extracellular space.

In our study, the *in vitro* experiment carried out on DID labeled cells allowed to obtain the reference point for further explants analysis. Cell number which can be successfully visualized with IVIS® as distinguishable to the eye of the recipient was semi-quantitatively specified. Furthermore, these results indicate that imaging using IVIS® is specific with respect to the far red dye-labeled cells. It is confirmed by the compliance of presented readings obtained with this system compared to microscopic technique, which ranks at 89%. For comparison, in the case of using the PKH26 similar compliance reached 0–35% depending on the visualization method. Thus, presented data supports the hypothesis of the such a method usefulness, but only for the DID dye. However, even in the case of DID, along with high specificity going limited sensitivity of the method (80%) in the material analyzed in the presented study. The reason for that is probably the relatively low own sensitivity of used device. This was confirmed by *in vitro* part of this work, where signal for both dyes disappeared at the cell number less than 0.25x10^6^ even when they were analyzed in pure PBS, which generates minimal background.

Another problem of the analysis is the use of external controls, which in imaging techniques is always burdened by an error. The best control during the imaging is internal control, which is the same tissue (or animal) prior to treatment. For intravital imaging of small rodents like mice to which IVIS® is dedicated, the animal can be tested before and after the administration of the labeled material. For cells grafting into the urethra or other organ in a large animal model the internal control is impossible to use, because of the animal size and the necessity to analyze the tissues or organs isolated post-mortem. The availability of tissue only after the end of the study period does not allow for an initial fluorescence measurement and determining the fluorophore to the background signal ratio. A partial solution could be provided in the future by conducting experiments on the urethras taken from animals that have not been previously injected with cells. It would be need to make raw tissue imaging, and then to inject labeled cells to the same urethra at various concentrations, at different depths of tissue, as to reconstruct accurately the course of the experiment. Further analysis of the injected tissue and calculating the ratio of signal originating from cells strength to the endogenous tissue fluorescence would allow for the improvement of the method sensitivity. However this would require the inclusion of more animals for experiments and would be limited by the duration of follow-up. Currently, the only *in vivo* imaging technique which allow tracking cells after transplantation in large animals is magnetic resonance imaging (MRI). Although magnetic resonance is a very good diagnostic tool in clinical terms, it has low sensitivity with regard to trace the fate of the cells following transplantation. The improvement of MRI usefulness in tracking cells after transplantation was one of the key objectives addressed by a team of researchers working on the EU project ENCITE ('European network for cell imaging and tracking expertise') [36]. Although novel solutions has been proposed and tested like using gadolinium or fluorine isotope (^19^F) instead of iron for cell labeling, this method is still rather utilized in small animal models [37, 38]. The use of this technology for large experimental animals in the field of regenerative medicine entails a lot of inconvenience. Firstly, the device of this type is not readily available to large animals and the use of the device intended for human patients is impossible. This type of equipment would have to be near the animal house in order to not transport animals, exposing them to additional stress. *Ex vivo* imaging system, used in the way presented in this paper, is used to analyze collected tissue without transporting the animals to the laboratory. Of course, this method limits the assessment only to the end-point of the study, but the analysis is easy, brief and enables screening the whole region of interest without affecting the tissue properties. The method is based on the widely used phenomenon of fluorescence. Labeling with fluorescent dyes is convenient, easy and generates moderate costs. Thus, the strategy seems to be appropriate to use in assessing the transplantation effects, especially in the study of transplantation to a particular tissue or organ, where multiorgan imaging is not necessary.

Used automatic spectral unmixing algorithm allows to extract searched signal from merged spectra. Cutting off the background derived from tissue autofluorescence or food fluorescence, gives a picture of transplanted cells fluorescence in the tested tissue [39]. In the presented study the impact of the animal nutrition methods for the background signal strength the has not been executed. It could represent some inconvenience. However exclusion from the diet of ruminants food containing chlorophyll is practically impossible, so the background signal remains at a certain level. In addition, using the fluorochrome with the far red range, where the influence of autofluorescence and fluorescence from food is insignificant, the issue of nutrition does not seem crucial. Therefore it is not necessary complicating the experiment including animals diet restrictions. It would be reasonable to take into account the aspect of diet to determine the transplantation effectiveness in explants from the digestive tract. In such a case, the influence of chlorophyll fluorescence would constitute a significant reduction in the use of the fluorophores excited by the waves shorter than 600 nm.

The proposed in this study method of tissue analysis is intended to allow a preliminary assessment of the transplanted cells location and eventually semi-quantitative analysis in the study group. It does simultaneously allow for further studies, including immunohistochemical evaluation. It should be mentioned that during the IHC staining difficulties associated with the fading of illumination of both used fluorophores were encountered. Visibility of cells was weakened or even underwent complete disappearance after staining for the presence of desmin, what confirmed previous reports [29, 40]. As the mentioned fluorophores are membrane dyes, each procedure interfering with the cell membrane continuity and increasing porosity, in order to improve the antibodies penetration in tissue, results in the release of fluorophores from cells and tissue. It can be observed by microscopic analysis of the specimen using a filter appropriate to the desired marker as increased background fluorescence. Elbelger et al. in their study demonstrated that the technique of specimens freezing and thawing did not produce this effect [40]. In the present study, the problem of illumination fading has been solved by using blocking solutions without detergents additives such as Triton X-100 and the reduction of tissue fixation time in cold acetone to a minimum (7 min). Obviously, only frozen sections were used as processing of paraffin embedded sections wash out the membrane lipophilic dyes.

The introduced data indicate that the *in vivo* imaging system such an IVIS® can be adapted as a method supporting evaluation of the cells transplantation effects in large experimental animals. The far red but not orange dyes for cell labeling are suitable for this purpose. This particular study was performed on isolated urethras, however, we claim that this way of analysis could be used also for other tissues or organs i.e. brain, spinal cord, heart or kidney. The method has a limited sensitivity, however, allows the assessment of the injected cells location while maintaining the entire tissue for further analysis. The detail histological evaluation can be performed on preselected areas. Taken together, the presented protocol improve and simplify studying of cell transfer effects in large animals. Moreover, it allows to reduce animal number and costs of the experiments in comparison to the traditional approaches.

## Conclusions

IVIS® system under appropriate conditions of the analysis and visualization can be used as a method for *ex vivo* tissue imaging. The far red dye in contrast to the orange dye is suitable to assess the transplantation effects of explants by using the IVIS®.

## Supporting information

S1 Fig Plate layout in *in vitro* experiment. A) 96-well plate with black walls and bottom prepared for the experiment- in rows E-H minced poultry muscle tissue; B) Schematic indication of the cell number in the wells (triplicates—grouped in the diagram); C, D) Plate layout in the experiment imaging cells using IVIS®.
(TIF)

S2 Fig *In vitro* experiment. **Plates visualized with IVIS**®. Images of DID (A, A’) and PKH26 (B, B’) labeled cells prior to (A, B) and after transfer to the wells with minced poultry muscle tissue (A’, B’).
(TIF)
