# Supplementary figures and images for: In vivo imaging system for explants analysis—A new approach for assessment of cell transplantation effects in large animal models
Source: PLoS One. 2017 Sep 20;12(9):e0184588. doi: 10.1371/journal.pone.0184588 (PMC5607129; doi:10.1371/journal.pone.0184588)

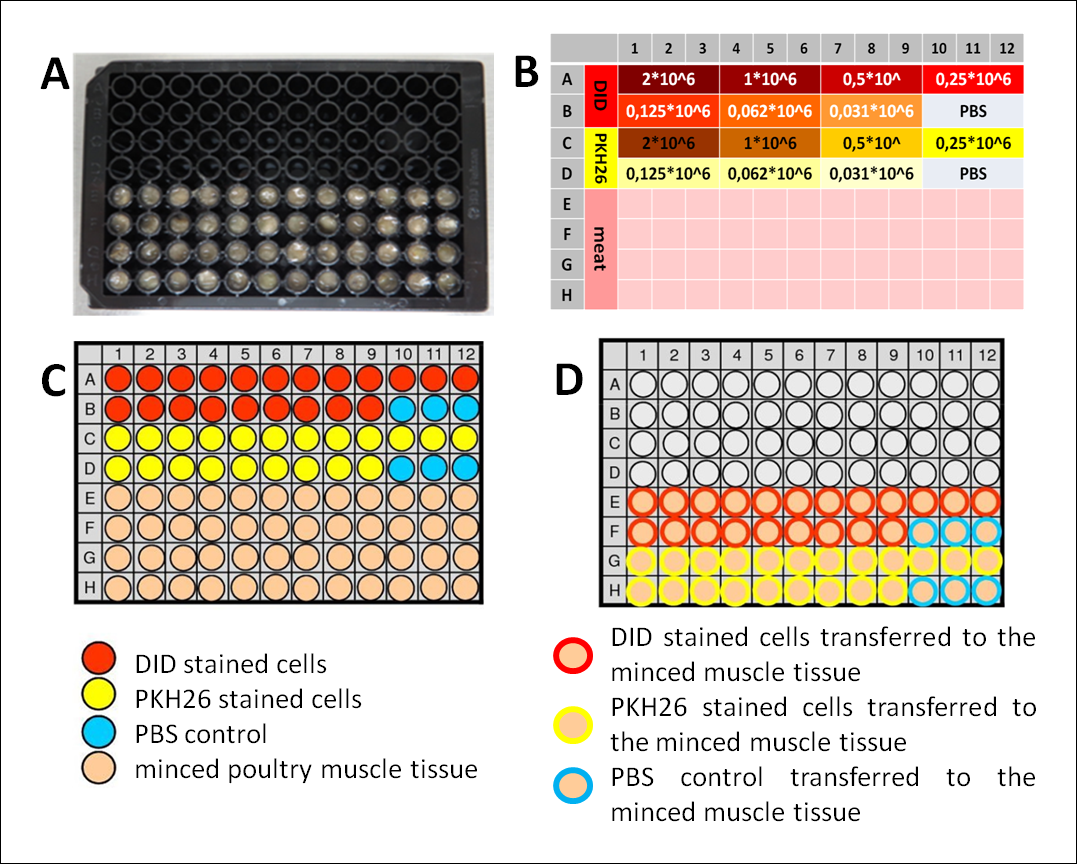

Supplement: S1 Fig — A) 96-well plate with black walls and bottom prepared for the experiment- in rows E-H minced poultry muscle tissue; B) Schematic indication of the cell number in the wells (triplicates—grouped in the diagram); C, D) Plate layout in the experiment imaging cells using IVIS®. (TIF) [file pone.0184588.s001.tif]

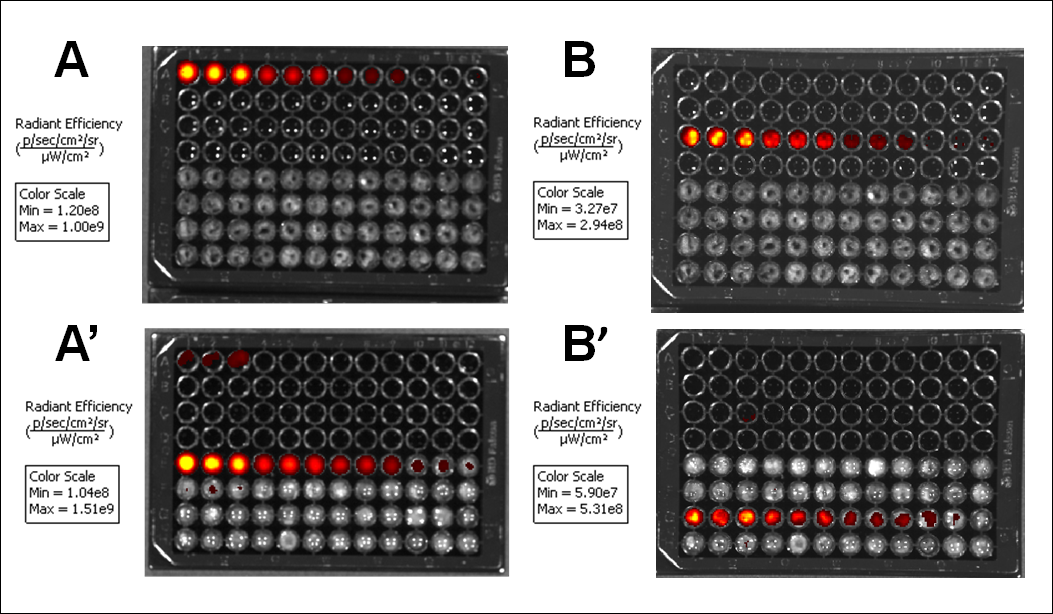

Supplement: S2 Fig — Plates visualized with IVIS®. Images of DID (A, A’) and PKH26 (B, B’) labeled cells prior to (A, B) and after transfer to the wells with minced poultry muscle tissue (A’, B’). (TIF) [file pone.0184588.s002.tif]
